# Food Allergy Training for Schools and Restaurants (The Food Allergy Community Program): Protocol to Evaluate the Effectiveness of a Web-Based Program

**DOI:** 10.2196/resprot.9770

**Published:** 2018-06-12

**Authors:** Inês Pádua, André Moreira, Pedro Moreira, Renata Barros

**Affiliations:** ^1^ Faculty of Nutrition and Food Sciences University of Porto Porto Portugal; ^2^ Faculty of Medicine University of Porto Porto Portugal; ^3^ Institute of Public Health University of Porto Porto Portugal; ^4^ Research Centre in Physical Activity, Health and Leisure University of Porto Porto Portugal; ^5^ Nutrition and Food Allergy Panel, Scientific Council of Economic and Food Safety Authority of Portugal Lisbon Portugal

**Keywords:** food allergy, eHealth, community, schools, restaurants

## Abstract

**Background:**

Food allergy is a growing public health concern. The literature suggests that a significant number of reactions occur in community services, such as schools and restaurants. Therefore, suitable training and education for education and catering professionals using viable and practical tools is needed.

**Objective:**

The objective of this study is to evaluate the effectiveness of a Web-based food allergy training program for professionals working in schools and restaurants, designed to improve knowledge and good practices in the community.

**Methods:**

Free learning programs which contain educational animated videos about food allergy were developed for professionals working at schools and restaurants. The learning programs comprise of nine 5-minute videos, developed in video animation format using GoAnimate, with a total course length of 45-60 minutes. The courses for professionals at both schools and restaurants include contents about food allergy epidemiology, clinical manifestations, diagnosis and treatment, dietary avoidance, emergencies, labelling, and accidental exposure prevention. Additionally, specific topics for work practices at schools and restaurants were provided. Food allergy knowledge survey tools were developed to access the knowledge and management skills about food allergy of school and restaurant staff, at baseline and at the end of the food allergy program. The courses will be provided on the e-learning platform of the University of Porto and professionals from catering and education sectors will be invited to participate.

**Results:**

Data collection will take place between September 2017 and October 2017, corresponding to a 2-month intervention. Final results will be disseminated in scientific journals and presented at national and international conferences.

**Conclusions:**

The Food Allergy Community Program intervention may improve school and restaurant professionals’ commitment and skills to deal with food allergy in the community. Furthermore, this e-intervention program will provide an innovative contribution to understanding the impact of electronic health technologies on the learning process and the development of strategies for community interventions.

**Registered Report Identifier:**

RR1-10.2196/9770

## Introduction

A food allergy is defined as a reproducible and specific immune response that occurs on exposure to a given food, leading to adverse health effects [1], affecting 2% to 5% of the population [2]. Currently, the primary treatment of food allergy is avoidance of the involved foods [1] but it is not uncommon for the lack of information to contribute to accidental dietary exposure [3]. Indeed, food allergy has emerged as a “second wave” of the allergy epidemic [4] and is now recognized as a public health problem, therefore requiring interventions at different societal levels. As a result, food allergy has been a problem under discussion in the legal and political sphere.

Studies show that a significant number of reactions occur in public places such as restaurants [5] and schools [6]. Additionally, they show that eating out is one of the key areas where food allergy patients feel there is a lack of information [7] and this results into an alarming feeling of insecurity when dining out. As a consequence of this insecurity, a fear of allergic reaction leads to patients limiting work, leisure, and social activities which include trips and meals, and this can have a wide impact on quality of life [8].

According to the literature, there is a consensus that there is a significant lack of information and education for the different stakeholders in the field of food allergy. Studies examining school personnel’s knowledge of food allergies have shown that teachers do not consider themselves sufficiently informed and prepared [9,10]. Moreover, the psychosocial impact of food allergies among school children is also a crucial and pressing issue. Several studies have shown an increase in cases of bullying with children with food allergy. A study by Lieberman et al found that 86% of food allergy pediatric patients in the United States of America report bullying [11] and, likewise, Muraro et al reported that, in Italy, 60% of children aged 8-19 years with a food allergy claimed that they were a victim of some form of bullying at least once in the past 2 months, a frequency which was twice as high than their nonallergic peers [12]. Thus, commitment and education of the stakeholders who provide food, deal with children and teenagers, and support their socialization are crucial for patients’ well-being in the school community [13].

When considering food allergy in restaurants, two studies have stated a particularly warring discrepancy between personnel’s knowledge about food allergy and their comfort level in providing a safe meal [14,15]. In a recent Environmental Health Specialists Network (EHS-Net) study on food allergy knowledge and attitude conducted by the Center for Disease Control and Prevention, the authors found that despite managers, food workers, and servers being generally knowledgeable about food allergies, critical knowledge gaps were present [16]. For example, more than 10% of the staff included in the study believed that a person with a food allergy can safely consume a small amount of the culprit allergen [16]. Additionally, staff reported a low confidence in their restaurants’ ability to correctly respond to a food allergy emergency [16].

When asked to identify barriers to learning about food allergies, restaurants’ personnel identified high training costs and time constraints as the main barriers [17]. School personnel reported that video and internet resources are the best tools for them to learn about food allergy [18]. A Web-based training program would overcome the constraints identified by restaurant personnel and satisfy the requirements for school personnel and therefore constitutes a viable and practical alternative for training professionals from different sectors [19].

Previous computer-based intervention programs used in both schools [6,20] and restaurants [21] pointed to the feasibility and effectiveness of a computer-based training; however, these interventions are scarce, suggesting the need for more research in this area. The main aim of this study is to evaluate the effectiveness of a Web-based food allergy training program for schools and restaurants, which will promote food allergy knowledge and awareness in catering and education staff.

## Methods

### Study Design

The Food Allergy Community (FAC) program is an intervention program which intends to evaluate the effectiveness of Web-based food allergy training on food allergy-related knowledge and practices of professionals in the education and catering sectors. The project began in January 2015 and data collection will occur in between September 2017 and October 2017.

### Participants and Data Collection

The target participants in this study are professionals in the catering and education sectors from Portugal, Portuguese-speaking African countries, and Brazil, who are interested in courses on food allergy management in restaurants and schools. The e-learning courses will be held on the University of Porto e-learning platform, *Academia UP*. The courses will be announced and widely disseminated through the media. Interested professionals in the catering and education industries will be asked to register through an online form, available at *Academia UP*, and provide their name, contact details, and occupation. All professionals who meet the inclusion criteria, namely teachers, educational assistants, health professionals, and tourism, hotel, and restaurant professionals who are currently employed in either the education or catering sector, as well as students, will be included in the study.

The e-learning platform is private, and access will be restricted by the research team providing individual credentials to the included participants. When participants first log in to the e-platform, they will be asked to read and complete the informed consent form in order to participate in the research. The consent form includes the purpose of the study, the level of data protection, and information on participants’ right to withdraw during the study. Additional instructional videos and materials will be available for participants to familiarize themselves with the online course procedures. After gaining informed consent, participants will be asked to complete a brief questionnaire on their demographic characteristics such as gender, region, educational background, occupation, and workplace specificities (eg, the classification of the restaurant or the school’s grade level).

After agreeing to join the study and acknowledging that the intervention includes both pre- and posttests to assess the participant’s knowledge, participants will be randomly assigned into any of the 3 following groups: (1) the pretest group, (2) the posttest group, or (3) the pre- and posttest group. The existence of 3 groups will allow for the measurement of a possible interactive effect between pretesting and the intervention.

### Ethics

This research was conducted according to the guidelines established by the Declaration of Helsinki, and the study protocol was approved by the Ethics Committee of University of Porto (24/CEUP/2016) and by the Portuguese National Commission of Data Protection (735/2017).

### Food Allergy Knowledge and Practice Survey Tools

Two different Food Allergy Knowledge survey tools (one for each sector) were developed after an extensive review of the literature to determine food allergy knowledge and management skills of school and restaurant staff. They were based on previous questionnaires for restaurant [16,22,23] and school [9,24] staff, as well as other educational materials [25-27]. Initially, the questionnaires were reviewed by a panel of food allergy experts to optimize readability and question clarity, and to ensure they were at an appropriate difficulty level. Both questionnaires were tested by representatives of the target groups in a pilot study. Some questions were adjusted to improve questionnaires’ validity, after which both questionnaires were converted to an online survey, therefore allowing the expected learning outcomes for the food allergy courses to be established (Textbox 1).

The questionnaires will be completed by the participants twice, before and after the training. The first questionnaire will assess the baseline level of knowledge of the target population which is important to generate a statistically valid baseline to determine the impact of the intervention. The questionnaire completed after completion of the e-learning modules will be used to determine the level of change in impact and outcome indicators between the baseline and final evaluations.

The first section of the questionnaire contains 20 multiple-choice questions with a single-best-answer (each scored one point if correct) to measure participants’ knowledge and skills related to food allergies. The first 11 questions are common to both surveys and evaluate six different dimensions: epidemiology, diagnosis, symptoms and treatment, dietary avoidance, emergency procedures, food labelling, and the food allergies legal framework. The last 9 questions are specific to the target group and are different in the two surveys. For the questionnaire specific for school personnel, these questions evaluate good practice at the workplace, in particular the prevention of accidental exposure and awareness of bullying. For restaurant personnel, the questions evaluate the good practice in all stages of customer service, considering the prevention of cross-contact.

To evaluate whether the knowledge and acquired skills meet the learning outcomes foreseen for the course (Textbox 1), the questions will have both a theoretical domain (general concepts about food allergy and allergen avoidance) and a practical domain (regarding procedures to be followed in practical situations).

The second section of the questionnaire (the section specific to each sector) corresponds to questions regarding work-related practices and perceived responsibilities.

**The expected learning outcomes of the food allergy courses.**

**Learning outcomes for school and restaurant staff**

- Understand the differences between food allergies, food sensitivities, and intolerances
- List the most common food allergens
- Recognize the symptoms of severe food allergic reactions and anaphylaxis
- Identify the diagnostic procedures of a food allergy
- Realize that an allergic reaction can be triggered not only by the consumption of the food but also by inhalation or skin contact
- Be aware that there is no routine treatment for food allergy, apart from dietary avoidance
- Be aware of how to avoid allergen exposure, including food labelling interpretation
- Respond safely and appropriately if an allergic reaction occurs
- Know the current legislation on the protection of the consumer with food allergy

**Learning outcomes specific for school staff**

- Prevent accidental exposure at school, namely in the canteen, classroom, playground, or bus
- Recognize bullying as one of the main psychosocial impacts of food allergy in children and teenagers

**Learning outcomes specific for restaurant staff**

- Communicate effectively with food-allergic customers to ascertain their needs
- Prepare and serve a safe meal to a food-allergic customer, considering the prevention of cross-contact

The responses will be addressed either through yes or no questions or a 5-point Likert-type scale, ranging from 1 (“Strongly disagree”) to 5 (“Strongly agree”), to examine whether personal experiences and training would impact food allergy risk perception and communication. After completing the Food Allergy Knowledge and Practice Survey Tools, participants will be able to join to the free online courses from the FAC Program.

### E-Learning Food Allergy Courses for Schools and Restaurants

The intervention consists of an online asynchronous training program, which comprises of several lectures in video format about general concepts related to food allergy and food allergy management in schools and restaurants.

The training modules were developed based on the European Academy of Allergy and Clinical Immunology guidelines [28] and the Food Allergy Portuguese Guidelines, namely Food Allergy in Schools [29] and Food Allergy in Restaurants [30], published by Directorate-General of Health, in partnership with the Faculty of Nutrition and Food Sciences and the Faculty of Medicine at the University of Porto. Scripts for the videos were first developed and then translated into a video animation format, using GoAnimate (GoPremium; GoAnimate Inc, San Mateo, CA, USA), allowing for greater dynamics within the video and for participant engagement. The total course comprises of 9 modules, which contains 9 videos approximately 5 minutes in length. Therefore, the average time for completing the training is 45-60 minutes.

After considering the learning needs of the target audience, it was determined that the courses should both have a theoretical and a practical component (Table 1). The first six modules are similar for both courses and the lecture topics include definitions and concepts regarding food allergy symptoms, prevention, diagnosis, and treatment. The last three modules are sector-specific, and address food safety and best practices in the workplace, covering questions regarding accidental exposure prevention and working tips.

The participants will have 4 weeks to complete the entire course (9 modules), and two to three modules will be launched per week. The platform does not restrict the number of times the modules are accessed during the course.

In addition to the course modules, the platform will also provide complementary readings and tools, including expert interviews and a live demonstration of how to use self-injectable epinephrine devices, as well as an interactive forum where participants can share their doubts and experiences. The participants who complete the course and obtain a grade higher than 9.5, out of 20 possible points, will receive a certificate of participation from the FAC Program.

### Statistical Analysis

After the food allergy Web-based intervention program for schools and restaurants has been completed, statistical analysis of scores’ improvement and differences between the three groups will be performed using SPSS Statistical Software (SPSS Inc, Chicago, IL, USA) and R software program (The R Foundation for Statistical Computing, Vienna, Austria).

Descriptive analysis (proportions) will be performed to characterize the participants and to summarize the answers to the Practice and Perceptions Survey Tools. To access the improvement on the participants’ knowledge, General Linear Model for Repeated Measures test will be performed, allowing the 3 intervention groups to be compared. A *t* test will be conducted to compare the knowledge score means of the initial evaluation between the pre- and posttest group and the pretest group and the knowledge score means of the final evaluation between the pre- and posttest group and the posttest group.

**Table 1:** Modules included in the Food Allergy Online Course for Schools and Restaurants.

Module number		Module name
**General modules**		
	1	Food allergy: General concepts
	2	Food allergy: clinical manifestations, diagnosis, and treatment
	3	Food allergen avoidance: Why it is so difficult?
	4	Emergencies: What can I do?
	5	Food allergen labelling
	6	Food allergy prevention: Is it possible?
**Modules specific for schools**		
	7	Food allergy in school I: Basics and canteen
	8	Food allergy in school II: Inside the classroom and playground
	9	Bullying and the psychosocial burden of food allergy
**Modules specific for restaurants**		
	7	Communication and client’s attendance
	8	Cooking for allergic clients and meal service
	9	Organization and storage: A helpful way to reduce the risk

Item response theory using the two-parameter logistic model will be performed to determine the quality (difficulty and discrimination) of the questions from the food allergy knowledge survey.

## Results

Data collection will take place between September 2017 and October 2017, corresponding to a 2-month intervention. Final results will be disseminated in scientific journals and presented at both national and international conferences. Additionally, after the results of the study have been obtained, the courses could be permanently integrated into the training offered by the University of Porto, providing an added value for the formation of the community.

## Discussion

Progress in e-learning and related technologies are creating the basis for developments in the education and teaching, with a gradual change from traditional and on-site teaching to a modern teaching, which is mainly Web-based [31].

In 2002, the United Nations Educational, Scientific and Cultural Organization (UNESCO) proposed Open Educational Resources, which are defined as “Teaching, learning, and research materials in any medium, digital or otherwise, that reside in the public domain or have been released under an open license that permits no-cost access, use, adaptation, and redistribution by others with no or limited restrictions” [32]. Beyond this concept, UNESCO also states that Open Educational Resources “provide a strategic opportunity to improve the quality of education as well as facilitate policy dialogue, knowledge sharing, and capacity building” [32]. Despite the influence of new information and communication technologies, educational interventions in the food allergy field are particularly scarce, and the effectiveness of training about food allergy has still mainly been demonstrated with face-to-face interventions.

Wahl et al [33] conducted a study where school personnel attended a training program which comprised of a 45-minute educational food allergy training presentation, which included key facts about food allergies, allergen avoidance, recognition of symptoms, and emergency treatment, and showed that the training program was an effective strategy for helping individuals who work with children to feel more confident in dealing with food allergies. In a similar study conducted by Polloni et al [9], an increase in school teachers’ food allergy knowledge was observed after attendance at free multidisciplinary food allergy courses, which also highlighted the need for specific educational interventions. The same effectiveness in improving teachers’ knowledge about food allergy was also found by Ravarotto et al in a study that presupposes the design, implementation, and monitoring of food allergy workshops [10]. Ultimately, the results of a study conducted by White et al [6], which aimed to evaluate the effectiveness of a computer-based learning module as an additional food allergy teaching tool for school staff, pointed to the feasibility and effectiveness of this computer-based training, despite it being non-interactive.

Regarding studies on food allergy interventions for restaurants, Bailey et al designed and evaluated a food allergy educational intervention consisting of a 1-hour lecture about food allergy basics, accidental exposure, food labelling, emergencies, and proper communication with costumers [34]. At the end of the intervention, the authors concluded that food allergy training improved participants’ food allergy knowledge and workplace practices. Considering the scarcity of studies in this field, we extended our literature search to related areas, for example, food safety programs in restaurants, aiming to review the methodology and results. Indeed, Dittmar et al developed a 2-hour online program to enhance the knowledge of food safety best practices which included scientific contents that met the criteria established by Texas Department of State Health Services (USA), along with interactive activities and handouts to reinforce key concepts in the program [21]. The authors concluded that the program was an effective tool to increase training outreach, given that there was a growing number of food service employees completing the course.

Finally, and more comprehensively, a review of massive open online courses related to health and medicine reported that there was potential to use massive open online courses to educate health care practitioners and students and to increase health literacy among the public [35].

Our study has some limitations, namely that (1) all participants require internet and basic computer knowledge, (2) the self-enrollment of the participants raises questions about the representativeness of the sample of participants and whether the participants are already interested and educated about food allergy, and (3) the higher possibility of drop-outs compared to on-site training. However, studies in both areas conclude that food allergy training and new information and communication technologies may be an effective tool to enhance knowledge in the fields of food allergy, food science, and food safety.

Additionally, the FAC Program will provide an innovative and important contribution to understanding the impact of new information and communication technologies on people's learning, which seems to be crucial for ongoing development of online training programs. Furthermore, it will provide more data on the basic knowledge of catering and education professionals, which is extremely useful since these data are scarce in Europe. The information on professionals' basic knowledge is not only useful for the continuity of the training in new editions of the program, but also for the development of better strategies for community interventions and new governmental and public health guidelines. Professionals may find that they can make use of this program as learning tool without great impact on their routine and budget, which could improve their commitment and their skills to deal with food allergies, leading to a greater awareness about food allergy and an increase on food allergic-patients’ safety and integration in the community.

## Abbreviations

EHS-Net: Environmental Health Specialists Network
FAC: Food Allergy Community
UNESCO: United Nations Educational, Scientific and Cultural Organization
